# Evaluating oxidative stress, serological- and haematological status of dogs suffering from osteoarthritis, after supplementing their diet with fish or corn oil

**DOI:** 10.1186/s12944-016-0304-6

**Published:** 2016-08-26

**Authors:** Stella Maria Barrouin-Melo, Johanna Anturaniemi, Satu Sankari, Mikko Griinari, Faik Atroshi, Sakaewan Ounjaijean, Anna Katrina Hielm-Björkman

**Affiliations:** 1 Department of Equine and Small Animal Medicine, Faculty of Veterinary Medicine, University of Helsinki, P.O. Box 57, 00014 Helsinki, Finland; 2 Department of Anatomy, Pathology and Clinics, School of Veterinary Medicine and Zootechny, Federal University of Bahia, Av. Adhemar de Barros, 500, CEP: 40170-110 Salvador, Bahia Brazil; 3 Clanet Oy, Lotankatu 1, 02680 Espoo, Finland; 4 Department of Pharmacology and Toxicology, Faculty of Veterinary Medicine, University of Helsinki, P.O. Box 57, 00014 Helsinki, Finland; 5 Rinnekoti Research Centre, Nousumäki 2, 02980 Espoo, Finland

**Keywords:** OA, Osteoarthritis, Dog, Natural model, MDA, GSH, NTBI, 8-OH-dG, Omega-3, Omega-6, Fatty acid

## Abstract

**Background:**

Oxidative stress plays an important role in the pathogenesis of disease, and the antioxidant physiological effect of omega-3 from fish oil may lead to improvement of canine spontaneous osteoarthritis (OA).

**Methods:**

In this prospective randomized, controlled, double-blinded study, we assessed haematological and biochemical parameters in dogs with OA following supplementation with either a concentrated omega-3 deep sea fish oil product or corn oil. Blood samples from 77 client-owned dogs diagnosed as having OA were taken before (baseline) and 16 weeks after having orally ingested 0.2 ml/Kg bodyweight/day of deep sea fish oil or corn oil. Circulating malondialdehyde (MDA), glutathione (GSH), non-transferrin bound iron (NTBI), free carnitine (Free-Car), 8-hydroxy-2-deoxyguanosine (8-OH-dG), and serum fatty acids, haemograms and serum biochemistry were evaluated. Differences within and between groups from baseline to end, were analysed using repeated samples *T*-test or Wilcoxon rank test and independent samples *T*-test or a Mann-Whitney test.

**Results:**

Supplementation with fish oil resulted in a significant reduction from day 0 to day 112 in MDA (from 3.41 ± 1.34 to 2.43 ± 0.92 μmol/L; *P* < 0.001) and an elevation in Free-Car (from 18.18 ± 9.78 to 21.19 ± 9.58 μmol/L; *P* = 0.004) concentrations, whereas dogs receiving corn oil presented a reduction in MDA (from 3.41 ± 1.34 to 2.41 ± 1.01 μmol/L; *P* = 0.001) and NTBI (from −1.25 ± 2.17 to −2.31 ± 1.64 μmol/L; *P* = 0.002). Both groups showed increased (albeit not significantly) GSH and 8-OH-dG blood values. Dogs supplemented with fish oil had a significant reduction in the proportions of monocytes (from 3.84 ± 2.50 to 1.77 ± 1.92 %; *P* = 0.030) and basophils (from 1.47 ± 1.22 to 0.62 ± 0.62 %; *P* = 0.012), whereas a significant reduction in platelets counts (from 316.13 ± 93.83 to 288.41 ± 101.68 × 10^9^/L; *P* = 0.029), and an elevation in glucose (from 5.18 ± 0.37 to 5.32 ± 0.47 mmol/L; *P* = 0.041) and cholesterol (from 7.13 ± 1.62 to 7.73 ± 2.03 mmol/L; *P* = 0.011) measurements were observed in dogs receiving corn oil.

**Conclusions:**

In canine OA, supplementation with deep sea fish oil improved diverse markers of oxidative status in the dogs studied. As corn oil also contributed to the reduction in certain oxidative markers, albeit to a lesser degree, there was no clear difference between the two oil groups. No clinical, haematological or biochemical evidence of side effects emerged related to supplementation of either oil. Although a shift in blood fatty acid values was apparent due to the type of nutraceutical product given to the dogs, corn oil seems not to be a good placebo.

## Background

Alongside the importance of knowledge of canine diseases in veterinary medicine, pet dogs have increasingly been used as translational models for human diseases such as osteoarthritis (OA).

The three joints predominantly affected in canine OA are the hips, elbows and knees [1]. OA commonly occurs as a consequence of hip and elbow dysplasia [2]. In these cases, even though the genetic background might be crucial for the development of joint mechanical instability, years of genetic selection by dog breeders has not reduced the incidence of the disease [3]. Factors such as nutritional imbalance [4, 5], chronic inflammation [6, 7], ageing [8, 9] and obesity [10, 11] are linked to the development of OA in dogs and in humans. These factors are also associated with oxidative stress [12]. Structural, cellular and molecular alterations due to proteoglycan breakdown and inflammation triggered by e.g. IL-1 are present in the evolution of OA [13].

Osteoarthritis is a condition that causes pain, inflammation and stiffness in many joints. Non-steroidal anti-inflammatory drugs (NSAIDs) and disease-modifying anti-rheumatic drugs (DMARDs) are available today for the treatment of inflammatory disorders; however, these drugs have side effects. NSAIDs react by blocking the activity of cyclooxygenases (COXs) during inflammation [14]. COX is a bifunctional enzyme exhibiting both COX and peroxidase activities [15]. While the COX component converts arachidonic acid (AA) to a hydroperoxy-endoperoxide, the peroxidase component reduces the endoperoxide to the corresponding alcohol, the precursor of prostanoids, such as thromboxanes and prostaglandins [16]. Many promising new treatment approaches for OA are available. The search for more natural anti-inflammatory agents that can selectively block the activity of COX-2 during inflammation is ongoing, with enhanced therapeutic effect and little or no side effects even with prolonged usage high on the wish list. Scientists have provided new evidence that fish oil supplementation decreases the formation of pro-inflammatory prostanoids, which, when produced in excess, increase inflammation in various tissues and organs [17, 18]. Fish oil is rich in docosahexaenoic acid (DHA) and eicosapentaenoic acid (EPA), n-3 fatty acids that act as competitive substrates for the enzymes of AA metabolism and its products [19].

Reactive oxygen species (ROS), associated with pro-inflammatory cytokines and prostaglandins, have been shown to play a deleterious role in the course of joint diseases, leading to cartilage damage [20, 21] and progressive chronic inflammation [22, 23]. Comparable changes in the synovia immune cellularity have been described in dogs as those occurring in humans [24, 25]. An increased level of oxidation in the synovial fluid of osteoarthritic joints has been observed [26, 27], and increased activity of antioxidant enzymes has been associated with decreased viscosity of the synovial fluid [28]. Also, markers of oxidative stress and levels of antioxidant enzymes change in the blood of human patients and dogs with OA. These include serum catalase (CAT), superoxide dismutase (SOD), glutathione (GSH) and malondialdehyde (MDA) [29, 30].

Lipid metabolism in tissues affected by OA includes peroxidation reactions that link inflammation, oxidative stress and cartilage/bone tissue damage [28]. Lipid peroxidation may be involved in the clinical consequences of pain and dysfunction in the joint [31, 32]. Thus, dietary lipid composition is an important issue. Fish oil supplements, rich in omega-3 polyunsaturated fatty acids (PUFAs), e.g. EPA, have been claimed to be beneficial in the treatment of diseases such as rheumatoid arthritis [33]. After fish oil supplementation, the omega-3 s EPA and DHA increased in both plasma and neutrophil membranes of human patients exhibiting clinical improvement of OA [34]. Supplementation of n-3 PUFA in cell culture studies as well as in animal in vivo studies showed that cell proliferation was inhibited by production of lipid peroxides [35, 36]. Omega-3-related inhibition of cell proliferation via lipid peroxidation is helpful in conditions such as cancer, although lipid peroxidation and formation of ROS are thought to produce tissue damage [28]. Under in vitro conditions using endothelial cells, omega-3 supplementation resulted in an indirect antioxidant effect, characterized by reduced formation of lipid peroxidation products associated with direct superoxide scavenging, thus reducing inflammation, as compared with supplementing with other long-chain PUFAs [37].

Evidence suggests that omega-3 supplementation benefits human patients [38–40] and dogs [41–44] suffering from OA. Earlier in vitro studies have reported some beneficial effects of omega-3 fatty acids on cartilage cell inflammation and metabolism [45, 46] that are consistent with clinical observations. In dogs, the quality and mechanisms underlying nutraceutical therapy with omega-3 oils are just beginning to be investigated.

This paper reports our findings on the circulating levels of fatty acids, oxidative stress markers and antioxidant molecules as well as blood counts and serum biochemistry as outcome measures in a controlled double-blind clinical trial designed to compare the effects of omega-3 fatty acids and corn oil in dogs suffering from OA.

## Methods

Data on the clinical parameters have been published earlier [43], with a detailed description of the study protocol, the inclusion criteria for dogs, the diet and the supplements and the blood sampling protocol. The materials and methods section here contains only a brief description of the dogs and study design.

### Dogs and ethics

Briefly, 77 dogs met the inclusion criteria of age of at least one year, a body weight exceeding 18 kg, predetermined clinical signs of OA, a Helsinki chronic pain index (HCPI) [47] of over 6 and a radiographic diagnosis of hip, knee or elbow OA. Exclusion criteria were neurological deficits, articular infection, recent trauma, pregnancy, lactation or the owner would not comply with changing the dogs’ diet or with giving the supplement on a daily basis. The dogs lived with their owners during the entire study period, and generally had low physical activity due to their OA condition. The owners of the dogs were instructed not to give non-steroidal anti-inflammatory drugs (NSAIDs) or other analgesics for the two weeks preceding the baseline visit if possible, and not to give medication containing Na-pentosan polysulphate for 30 days. They were also required to sign an informed consent form acknowledging that they could leave the study at any time, without giving a reason. The study protocol was approved by the Committee of Ethics of the University of Helsinki and was conducted at the Small Animal Hospital of the same University.

### Study design and test products

The study was run as a double-blind, randomized clinical trial with a treatment group (an omega-3 supplement comprising a concentrated oil product from deep sea fish) and a negative control group (corn oil), using the CONSORT guidelines [48]. All dogs were evaluated by radiographs, submitted to blood sampling, and assigned to groups using a stratified computer-generated four-block random list [43] using the HCPI pain severity level and the diet as strata. The groups were statistically equal in total number of dogs, gender (also whether castrated, sterilized or intact), mean body weight, mean age and mean disease (OA) duration in months at baseline [43]. All eligible dogs were started on a diet of commercial dry food, which was either a basic wheat/beef (Royal Canin® Croc) or a rice/lamb sensitive formula containing no wheat, soy, beef, preservatives or artificial colorants (Jahti & Vahti®). Both foods had similar nutritional composition with no fish oil-derived ω-3 fatty acids and a ω-6/ω-3 ratio of 11:1. Neither food contained ingredients with a known positive effect on OA.

**Fig. 1:**
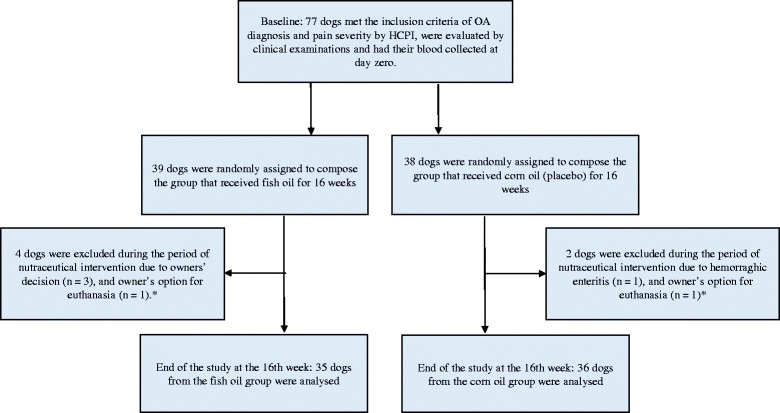
Flow chart showing the design and inclusion/exclusion criteria of the prospective study of fish oil as a nutraceutical intervention in dogs suffering from osteoarthritis (OA). Corn oil was compared with fish oil as placebo. *Information on the dogs’ clinical follow up and pain outcome are described by Hielm-Björkman et al. [43]

At baseline, the dogs were evaluated clinically, as described elsewhere [43], and had their blood samples collected. The dogs started the supplement trial by receiving a pharmaceutically cleaned dense deep sea fish oil with added vitamin E (Doils® Joints, Nutraceuticoils, Belgium) or corn oil (control) daily at the dose of 0.2 ml/Kg of body weight (BW) *per os*. Fish oil used in this study was concentrated by molecular distillation with a total omega-3 content of 63.6 % (Table 1). The fish oil contained 450 mg of EPA, 100 mg of DHA and 27 mg of eicosatetraenoic acid (ETA) per ml, with a total ω-3 content of 63.6 % of the total fatty acid content (Table 1). The same manufacturer supplied the corn oil with fish smell in identical containers to be used as a control. The corn oil contained only 1 % of ω-3, mainly α-linolenic acid (ALA), and no EPA, DHA or docosapentaenoic acid (DPA) [43]. To avoid oxidation of the fatty acids all dog owners were instructed to keep the oil supplement with the cap tightly shut in a dark refrigerator at 4-8 °C. At the end of the trial, at week 16, the dogs were again clinically evaluated [43] and blood samples were taken after an overnight fast. All outcome evaluators (veterinarians and owners) and technical assistants were blinded (Fig. 1).

**Table 1:** Composition of fish oil and corn oil products used as nutraceutical supplements for the study dogs suffering from osteoarthritis

Component	Fish oil g/100 g	Corn oil g/100 g
Analyzed fatty acids		
Palmitic acid (PA), C16:0^a^	0.6	10.2
Stearic acid (SA), C18:0^a^	4.6	1.8
Oleic acid (OA), C18:1 ω-9^b^	9.7	28.6
Linoleic acid (LA), C18:2 ω-6^c^	1.3	51.8
Gamma-Linolenic acid (GLA), C18:3 ω-6^c^	0.7	0.1
Eicosadienoic acid (EDA), C20:2 ω-6^c^	0.3	0
Dihomo-gamma-linolenic acid (DHGLA), C20:3 ω-6^c^	0.4	0
Arachidonic acid (AA), C20:4 ω-6^c^	2.1	0
Palmitoleic acid (PO), C16:1 ω-7^b^	0.2	0.1
α-linolenic acid (ALA), C18:3 ω-3^c^	0.6	1.0
Stearidonic acid (SDA), C18:4 ω-3^c^	2.4	0
Eicosatetraenoic acid (ETA), C20:4 ω-3^c^	1.8	0
Eicosapentaenoic acid (EPA), C20:5 ω-3^c^	43.9	0
Docosapentaenoic acid (DPA), C22:5 ω-3^c^	2.2	0
Docosahexaenoic acid (DHA), C22:6 ω-3^c^	11.4	0
Other fatty acids combined	17.8	6.4
%		
ω-3 fatty acids	63.6	1.0
ω-6 fatty acids	4.6	51.8
Ratio ω-6 / ω-3	0.07	52.2
Total SAFA^a^	6.4	12.8
Total MUFA^b^	16.4	29.5
Total PUFA^c^	69.5	53.2

^a^ *SAFA* saturated fatty acid, ^b^ *MUFA* monounsaturated fatty acid, ^c^ *PUFA* polyunsaturated fatty acid; Source: Hielm-Björman et al. (2012) [43], with a few additions

### Fatty acid analysis

The fatty acid composition of the oil products used was analysed by an international food testing laboratory using a modified AOCS Ce 1c-89 method (Eurofins, Raisio, Finland).

The fatty acid profile of the serum total lipids was analysed according to Seppänen-Laakso et al. [49]. Briefly, the serum lipids were first extracted with chloroform:dimethyl ether (2:1), then centrifuged at 3500 rpm. The lower chloroform layer was separated and evaporated overnight. Petroleum ether (0.5 ml) was added. The methyl esters were formed by adding 1 ml of 0.5 M Na-methylat and keeping the substance in a 50 °C water bath for 10 min. The samples were neutralized with 1 ml of NaHSO4, and the fatty acid methyl esters were extracted by adding 0.5 ml of petroleum ether. The samples were shaken for 10 min and centrifuged at 3500 rpm. The petroleum ether layer was analysed by DANI GC 1000 gas chromatography with NB-351 0.32 mm × 25 m capillary column by HNU-Nordion Ltd. (Helsinki, Finland). The column oven was programmed from 120 °C to 236 °C at 5.9 °C/min. Identification was based on retention times. Dietary fatty acid content was used to evaluate the adherence of each owner to providing the dogs’ respective supplement of fish oil or corn oil.

### Blood counts and biochemical analysis

Venous blood samples from overnight fasting dogs were taken at all three visits from the cephalic vein prior to any clinical tests or sedation. Whole-blood EDTA samples were immediately used for blood counts. To obtain plasma, part of the blood samples were taken in polypropylene tubes containing EDTA or Li-heparin and centrifuged at 1000 × g for 15 min. For serum, the blood was allowed to clot at room temperature for 30 min and then centrifuged at 1000 × g for 15 min. All serum and plasma samples were stored at −80 °C until analysed.

Complete blood counts were performed with an automated haematology multiparameter analyser adjusted for animal-cell counting (Cell-Dyn 3700 System, ABBOTT Diagnostics Division, ABBOTT Park, IL, USA). Blood smears were stained with May-Grünwald-Giemsa, and manual leucocyte differentials were determined by counting 200 cells.

The activity of serum alanine aminotransferase (ALT, EC 2.6.1.2 was measured according to the recommendations of the International Federation of Clinical Chemistry [50] and serum alkaline phosphatase (ALP, EC 3.1.3.1) activity according to the recommendations of the Scandinavian Society for Clinical Chemistry and Clinical Physiology [51]. Spectrophotometric methods were used for the determination of serum albumin, cholesterol, creatinine, glucose, total protein, triglycerides and urea. Analyses were performed by using a clinical chemistry analyser (Konelab 30i, Thermo Fisher Scientific, Vantaa, Finland).

Lipid peroxidation was determined by HPLC-based TBARS modified assay [52] and reported as MDA-equivalent. In brief, the malondialdehyde-thiobarbituric acid complex formed under high temperature (100 °C) and acidic conditions were injected onto the Spherisorb ODS2 (C18 column); 5 μm, 250 × 3.2 mm with guard column (C8) and eluted with 65 %, 50 mM potassium phosphate buffer pH 7.0 and 35 % methanol at a flow rate of 1.0 ml/min and detected at a wavelength of 532 nm.

Blood reduced GSH was determined using Ellman’s reagent [53] and served/applied as a marker of the antioxidant capacity of the organism. Briefly, an aliquot (0.2 ml) of whole blood was combined with 10 % sulphosalicylic acid. After centrifugation at 12,000 rpm for 2 min in a Eppendorf centrifuge at 4 °C, the supernatant was analysed spectrophotometrically at 412 nm, with 5,5′-dithiobis (2-nitro benzoic acid) in 0.1 M phosphate buffer, pH 8.0, for non-protein thiols.

Concentrations of 8-hydroxy-2-deoxy Guanosine (8-OH-dG) were measured in serum samples by an 8-hydroxy-2-deoxy Guanosine EIA kit (Cayman Chemical, Ann Arbor, MI, USA) to evaluate oxidative DNA damage and used as an indicator of oxidative stress [54].

Serum free iron (NTBI) concentration was measured by NTA-chelation/HPLC-based assay principally described elsewhere [54, 55] and used as a surrogate marker of iron metabolism and oxidation in the body.

Free carnitine (Free-Car) was measured using a Konelab 20XTi analyser (ThermoFisher Scientific, Vantaa, Finland) and used as a marker of antioxidant capacity of the body. Serum samples were pipetted to tubes (Centrifree Amicon, Gloucestershire, UK), which were centrifuged at 2000 g for one hour. Clear supernatants were used in assays of free carnitine. For determination of total carnitine, this supernatant was alkalized with 1 M NaOH. The incubation time at 60 °C was one hour. After cooling the sample was acidified with 2 M HCl. This sample was used for analysis of total carnitine. Carnitine reacted with acetyl-CoA to form acetylcarnitine and CoA. Carnitine acetyltransferase was the catalyser in this reaction. Free CoA reacted then with DTNB (5, 5-dithiobis (2-nitro benzoicacid)). Forming a complex, the thiophenolate ion was then measured at 405 nm. The detection limit was 2.0 μmol/L.

### Statistical analysis

All 77 intended-to-treat dogs were analysed. To calculate differences within groups from baseline to end, a repeated samples *T*-test and Wilcoxon rank test were used if the variables were normally distributed or not, respectively. To test differences between groups at the different evaluation times, an independent samples *T*-test or a Mann-Whitney test was used, using the same criteria of normality.

## Results

### Blood concentrations of fatty acids

Dogs were supplemented for 16 weeks with fish oil (*N* = at start 39/at end 35) or corn oil (*N* = 38/36). No significant differences between groups in terms of dogs per group, gender, age, body weight or disease severity/body distribution were observed [43].

As expected, serum concentrations of different fatty acids reflected the fatty acid composition in the nutraceutical products. All in all 69.5 % of the total fat content of the fish oil comprised PUFAs, whereof 63.6 % omega-3 and 4.6 % omega-6 fatty acids. The fat content of the corn oil had 52.8 % PUFAs, 1.0 % omega-3 and 51.8 % omega-6 fatty acids. The omega-6/omega-3 ratio of the fish oil and the corn oil was 0.07 and 51.8, respectively [43].

At the beginning of the trial, all dogs randomly distributed in either group had statistically similar blood concentrations of each evaluated fatty acid (Table 2). The fatty acid blood concentrations at the end of the trial showed that the dogs from each group had received the intended nutraceutical product. The dogs that had received fish oil exhibited a significant rise in blood levels of the omega-3 fatty acids EPA, DPA and DHA (all with *P* < 0.001) and a reduction in the concentrations of omega-6 gamma-linolenic acid (GLA), AA and linoleic acid (LA) (all with *P* < 0.001) and palmitoleic acid (PO) (*P* = 0.004) at 16 weeks. A significant difference was also found in the blood concentrations of the same fatty acids between dogs from the fish and corn oil groups at the end of the trial, with dogs in the former group having higher EPA, DPA and DHA concentrations and lower GLA, AA and LA concentrations (all with *p* ≤ 0.001).

**Table 2:** Blood concentrations of fatty acids at baseline and at 16 weeks in dogs receiving fish oil or corn oil supplementation

Variable	Fish oil			Corn oil			Between groups	
	Baseline	End of trial	*P*-value/direction	Baseline	End of trial	*P*-value/direction	Baseline *P*-value	End *P*-value
Fatty acids, %								
PA, C16:0	15.44 ± 1.38	14.99 ± 1.88	0.191	**14.99 ± 1.31**	**14.60 ± 1.28**	**0.002 ↓**	0.183	0.336
SA, C18:0	21.42 ± 1.49	21.38 ± 1.65	0.891	21.87 ± 1.62	21.85 ± 1.67	0.919	0.246	0.252
OLA, C18:1 ω-9	9.57 ± 1.43	9.43 ± 2.41	0.758	9.13 ± 1.23	8.90 ± 1.14	0.243	0.180	0.255
PO, C16:1 ω7	**0.99 ± 0.35**	**0.85 ± 0.30**	**0.004 ↓**	0.91 ± 0.28	0.87 ± 0.25	0.407	0.339	0.739
LA, C18:2 ω-6	**22.97 ± 2.36**	**20.91 ± 2.26**	**<0.001 ↓**	**23.17 ± 2.60**	**24.13 ± 2.79**	**0.007** ↑	0.743	**<0.001** ^**b**^
GLA, C18:3 ω-6	**0.19 ± 0.07**	**0.13 ± 0.05**	**<0.001 ↓**	**0.17 ± 0.07**	**0.20 ± 0.10**	**0.018** ↑	0.322	**0.001** ^**b**^
EDA, C20:2 ω6	0.32 ± 0.09	0.31 ± 0.09	0.545	0.37 ± 0.08	0.36 ± 0.08	0.625	0.065	**0.044** ^**b**^
DHGLA, C20:3 ω-6	0.88 ± 0.19	0.81 ± 0.27	0.111	**0.95 ± 0.24**	**0.89 ± .25**	**0.011 ↓**	0.246	0.223
AA, C20:4 ω-6	**19.48 ± 2.26**	**14.2 ± 2.06**	**<0.001 ↓**	**19.48 ± 2.59**	**20.15 ± 2.51**	**0.030** ↑	0.995	**<0.001** ^**b**^
ALA, C18:3 ω-3	0.33 ± 0.11	0.31 ± 0.08	0.353	0.33 ± 0.14	0.34 ± 0.28	0.919	0.971	0.632
EPA, C20:5 ω-3	**0.98 ± 0.48**	**6.81 ± 2.96**	**<0.001** ↑	**1.04 ± 0.44**	**0.70 ± 0.31**	**<0.001 ↓**	0.633	**<0.001** ^**a**^
DPA, C22:5 ω-3	**2.07 ± 0.45**	**3.11 ± 0.99**	**<0.001** ↑	**2.16 ± 0.62**	**1.96 ± 0.57**	**0.004 ↓**	0.529	**<0.001** ^**a**^
DHA, C22:6 ω-3	**1.52 ± 0.59**	**3.00 ± 1.04**	**<0.001** ↑	**1.56 ± 0.71**	**1.30 ± .50**	**0.006 ↓**	0.790	**<0.001** ^**a**^

*P*-values for changes within and between groups; significant (*P* < 0.05) values bolded. ^a^Higher values in the fish oil group; ^b^Higher values in the corn oil group. See Table 1 for fatty acid abbreviations. ↑ Value increased significantly; ↓ Value decreased significantly

Conversely, dogs in the corn oil group showed a significant elevation in the blood concentrations of the omega-6 fatty acids GLA (*P* = 0.018), AA (*P* = 0.030) and LA (*P* = 0.007) and a decrease in omega-3 EPA (*P* < 0.001), DPA (*P* = 0.004) and DHA (*P* = 0.006) (Table 2). Reductions in Palmitic acid (PA) (*P* = 0.002) and omega-6 dihomogammalinolenic acid (DHGLA) (*P* = 0.011) serum concentrations were also observed within the corn oil group, but no significant difference was seen in the comparison between supplement groups at the end of the trial. The corn oil group showed higher concentrations of omega-6 eicosadienoic acid EDA (*P* = 0.044) at 16 weeks than the dogs that had received fish oil. No side effects were reported or seen in either group.

### Blood cell counts and biochemical analytes

At baseline, all blood parameters were statistically similar among all dogs (Table 3). The haemograms revealed some significant changes within and between the groups of dogs receiving fish or corn oil from the beginning to the end of the trial, although most values remained within the reference ranges (Table 3). The Mean Corpuscular Haemoglobin (MCH) and the Mean Corpuscular Haemoglobin Concentration (MCHC) values were slightly below the normal ranges and increased in dogs from both groups (fish oil: *P* = 0.003 and *P* < 0.001, corn oil: *P* < 0.001 and *P* < 0.001, respectively) during the 16-week trial. A significant reduction in the Mean Corpuscular Volume (MCV) (*P* = 0.010) was found only within the fish oil group. No significant difference was found in the comparison between groups.

**Table 3:** Biomarkers for lipid metabolism, oxidation and inflammation; blood counts and biochemistry per group at start (baseline) and end (16 weeks) of fish oil or corn oil trial

Variable	Fish oil			Corn oil			Between Groups	
	Baseline	End of trial	*P*-value/direction	Baseline	End of trial	*P*-value/direction	Baseline *P*-value	End *P*-value
Oxidation marker								
MDA (μmol/l)	**3.41 ± 1.34**	**2.43 ± 0.92**	**<0.001 ↓**	**3.41 ± 1.34**	**2.41 ± 1.01**	**0.001 ↓**	0.957	0.926
GSH (mmol/l)	1.97 ± 0.22	2.02 ± 0.17	0.200	2.01 ± 0.21	2.07 ± 0.22	0.184	0.645	0.296
8-OH-dG (pg/ml)	1.54 ± 0.60	1.64 ± 0.74	0.315	1.68 ± 0.60	1.75 ± 0.79	0.410	0.340	0.545
NTBI (μmol/l)	−1.36 ± 4.11	−1.92 ± 2.58	0.248	**−1.25 ± 2.17**	**−2.31 ± 1.64**	**0.002↓**	0.950	0.445
FCar (μmol/l)	**18.18 ± 9.78**	**21.19 ± 9.58**	**0.004** ↑	20.53 ± 9.78	22.23 ± 10.12	0.121	0.222	0.657
Haemograms (Unit) RV = Reference value								
Haematocrit % RV = 38–57	50.15 ± 4.24	47.55 ± 8.95	0.096	49.22 ± 5.15	48.38 ± 5.88	0.237	0.729	0.881
Haemoglobin (g/l) RV = 140–203	164.23 ± 14.00	162.57 ± 15.19	0.453	160.41 ± 16.71	160.94 ± 19.23	0.816	0.616	0.409
MCV (fl) RV = 67–80	**69.17 ± 2.15**	**68.73 ± 2.23**	**0.010 ↓**	69.90 ± 3.11	69.76 ± 2.54	0.636	0.339	0.180
MCH (pg) RV = 24–29	**22.64 ± 0.77**	**22.88 ± 0.87**	**0.003** ↑	**22.76 ± 0.95**	**23.20 ± 0.97**	**<0.001** ↑	0.572	0.494
MCHC (g/l) RV = 345–367	**327.33 ± 3.96**	**332.80 ± 5.93**	**<0.001** ↑	**325.81 ± 6.18**	**332.56 ± 6.79**	**<0.001** ↑	0.379	0.364
Platelet (n × 10^9^/l) RV = 102–395	304.76 ± 88.37	309.83 ± 98.56	0.692	**316.13 ± 93.83**	**288.41 ± 101.68**	**0.029 ↓**	0.991	0.513
RBC (n × 10^12^/l) RV = 5.3–8.0	7.25 ± 0.61	7.11 ± 0.74	0.198	7.04 ± .76	6.94 ± 0.84	0.319	0.474	0.291
WBC (n × 10^9^/l) RV = 5.4–17.4	8.37 ± 2.27	8.20 ± 2.36	0.564	8.45 ± 3.22	8.51 ± 3.31	0.913	0.712	0.975
Neutrophil % RV = 40–80	68.97 ± 10.52	66.46 ± 14.40	0.350	69.89 ± 11.30	70.56 ± 8.44	0.644	0.971	0.195
Lymphocyte % RV = 10–36	18.10 ± 8.61	19.68 ± 7.74	0.109	16.11 ± 7.20	16.82 ± 5.88	0.482	0.504	0.166
Monocyte % RV = 0–13	**3.84 ± 2.50**	**1.77 ± 1.92**	**<0.001 ↓**	**4.11 ± 3.02**	**3.06 ± 2.70**	**0.050 ↓**	0.340	**0.030** ^**a**^
Eosinophil % RV = 2–10	7.59 ± 3.53	9.01 ± 4.79	0.069	8.54 ± 5.33	8.42 ± 6.00	0.927	0.605	0.551
Basophil % RV = 0–1	**1.47 ± 1.22**	**0.62 ± 0.62**	**0.001**	1.33 ± 1.08	1.13 ± 0.96	0.396	0.882	**0.012** ^**a**^
Blood biochemistry								
Urea (mmol/l) RV = 2.4–8.8	5.23 ± 1.61	5.48 ± 1.31	0.308	5.12 ± 1.37	5.25 ± 1.80	0.695	0.430	0.536
Creatinine (μmol/l) RV = 57–116	**86.85 ± 9.36**	**90.65 ± 11.73**	**0.008** ↑	**88.62 ± 13.34**	**93.51 ± 14.85**	**0.001** ↑	0.372	0.311
ALP (U/l) RV = 33–215	127.83 ± 143.75	146.85 ± 170.32	0.052	147.32 ± 107.88	153.70 ± 116.36	0.283	0.598	0.843
ALT (U/l) RV = 18–77	52.35 ± 24.24	77.22 ± 136.25	0.279	58.60 ± 26.96	58.12 ± 28.77	0.888	0.452	0.414
Albumin (g/l) RV = 30–41	33.68 ± 2.02	33.77 ± 2.57	0.790	32.80 ± 2.39	33.24 ± 2.51	0.160	0.129	0.385
Total protein (g/l) RV = 58–77	63.53 ± 4.07	64.42 ± 4.34	0.158	63.97 ± 3.18	64.50 ± 3.59	0.469	0.990	0.932
Glucose (mmol/l) RV = 4.0–6.4	5.22 ± 0.38	5.34 ± 0.43	0.171	**5.18 ± 0.37**	**5.32 ± 0.47**	**0.041** ↑	0.645	0.854
Cholesterol (mmol/l) RV = 3.7–9.8	6.51 ± 1.90	6.94 ± 2.40	0.109	**7.13 ± 1.62**	**7.73 ± 2.03**	**0.011** ↑	0.463	0.135
Triglycerides (mmol/l) RV = 0.5–1.1	0.79 ± 0.37	0.71 ± 0.46	0.406	0.80 ± 0.31	0.81 ± 0.36	0.854	0.299	0.335

*MCV* Mean Corpuscular Volume, *MCH* Mean Corpuscular Haemoglobin, *MCHC* Mean Corpuscular Haemoglobin Concentration, *RDW* Red Cell Distribution Width, *RBC* Red Blood Cell Count, *WBC* White Blood Cell Count, *ALP* Alkaline Phosphatase, *ALT* Alanine Aminotransferase. *P*-values for changes within and between groups; significant (*P* < 0.05) values bolded. ^a^Lower counts in the Fish oil group; ^b^Higher values in the Corn oil group. *RV* Reference value. ↑ Value increased significantly; ↓ Value decreased significantly

A significant reduction in monocyte counts in dogs from both the fish oil (*P* < 0.001) and corn oil (*P* = 0.050) groups between baseline and 16 weeks was observed. Despite the reduction in monocyte percentage in both groups, there was also a significant difference between the groups (*P* = 0.030), so that the decrease was greater in the fish oil group.

Basophil counts, obtained by manual counting, were reduced significantly within the group of dogs receiving fish oil (*P* = 0.001) from the beginning to the end of the study, but not within the corn oil group. The comparison between groups also revealed that the basophil counts were significantly lower in the fish oil group (*P* = 0.012) than in the corn oil group at the end of the trial.

The platelet counts were significantly reduced within the corn oil group (*P* = 0.029) from the beginning to the end of the study. By contrast, in the fish oil group the platelets were increased, albeit not significantly.

The creatinine values increased in the serum of dogs from both groups (fish oil: *P* = 0.008, corn oil: *P* = 0.001) from the beginning to the end of the study. The final mean creatinine value was higher in the corn oil group, although the difference was not significant.

The group of dogs that received corn oil presented a significant elevation in serum values of glucose (*P* = 0.041) and cholesterol (*P* = 0.011), whereas the increases in the fish oil group were not significant. At the end of the trial, no statistical difference emerged between groups for either glucose or cholesterol serum values.

### Oxidative stress markers

The biomarkers that were chosen to reflect oxidative stress (MDA, 8-OH-dG and NTBI) and antioxidant capacity of the body (GSH and Free-Car) were statistically similar in the two dog groups at the beginning of the trial (Table 3). MDA values exhibited a significant reduction in dogs within both groups (fish oil: *P* < 0.001, corn oil: *P* = 0.001) from the beginning to the end of the trial. The comparison between groups did not reveal a statistical difference at the end of the study.

Serum values for 8-OH-dG exhibited a discrete and non-significant elevation within both groups. When these values were compared between groups, the mean value for the corn oil group was slightly higher than for the fish oil group, although the difference did not reach significance.

Serum free iron, in the form of non-transferrin bound iron (NTBI), resulted in negative values. Concentrations were higher among dogs receiving corn oil than among those receiving fish oil. The values for serum NTBI exhibited a decrease in both groups, but the reduction was significant only in the corn oil group (*P* = 0.002). No significant difference was found between groups at the end of the trial.

Blood GSH (mg/L) mean values exhibited minor (non-significant) elevations within both groups from the beginning to the end of the trial. The comparison between groups also did not reveal significant differences at the end of the trial.

Serum Free-Car values increased in both dog groups from the beginning to the end of the trial, but the difference was significant only in dogs within the fish oil group (*P* = 0.004). No difference was found between groups at any time.

## Discussion

Dietary supplementation of dogs suffering from OA with fish oil and corn oil clearly resulted in changes in blood concentrations of fatty acids, reflecting the fatty acid composition of the supplements. In a previous study, authors have reported a similar effect on blood profile of fatty acids in healthy dogs undergoing dietary trials with standard fish and corn oils [56]. Thus, the usage of oils for nutraceutical purposes, by the oral route, will promptly promote the bioavailability of fatty acids to the target tissues via the bloodstream. Both oils used in the trials were well tolerated and did not produce any adverse effects in the dogs over the 16 weeks; neither in clinical follow-up, haemograms or serum biochemistry for liver and renal function.

So far, the best clinical tools to definitively diagnose, classify and define disease progression or prognosis of OA in dogs are radiography, pain intensity measurements and functional assessments [57]. Nevertheless, such methods are sensitive only when the disease reaches an advanced stage, often with irreversible lesions in cartilage and bone [58]. A number of potential biomarkers have been studied to aid diagnosis, particularly in the early phase, and consensus exists that combined molecules could be applied for the assessment of disease outcome after the use of disease-modifying drugs [58]. A lipidomic approach has suggested that lipid metabolism can be altered during the course of OA in people, and an association between AA release from phospholipids due to an increased activity of phospholipase A2 (PLA2) and inflammatory pain has been described [59]. At least one clinical study has shown a relation between higher blood concentrations of AA, higher blood concentrations of omega-6 PUFAs and synovitis in patients with OA [39].

In this study in dogs, supplement-related changes in the serum fatty acid profile also influenced the associated metabolites. Fish oil supplementation induced a reduction in the serum concentration of LA and therefore also in the metabolites GLA, DHGLA and AA. Although the fish oil preparation used in our study contained 2 % AA, there was a pronounced decrease in serum AA concentration in the fish oil-supplemented dogs. A decrease in the AA concentration is a result of competition between AA and long-chain omega-3 PUFA for incorporation into circulating blood and tissue phospholipids [60]. Similar changes over a long period would be expected also in the phospholipids of other cells’ membranes. Long-chain PUFA omega-3 fatty acids (EPA, DPA and DHA) increased among serum total lipids, consistent with their concentrations in the supplemented fish oil. Supplementation with corn oil increased the LA content among serum total lipids and produced small but consistent increases in LA’s metabolites (EDA, GLA, DHGLA and AA). Although there was a significant difference in omega-6 EDA between the two groups at the end of the trial, this seems to be mostly due to the near-difference between the two groups already at baseline. EDA was also shown to be able to modulate the metabolism of PUFAs and alter the responsiveness of macrophages toward inflammatory stimulation [61]. However, the amelioration of functional parameters in both groups of dogs seen in the clinical data published earlier [43] suggests that not only the fish oil but also the corn oil produces some beneficial effect on the pathological condition. Despite not always reaching statistical significance, the global results of clinical and functional tests [43], together with the laboratory data obtained in this study, indicate a more consistent improvement in the dogs receiving fish oil than in the ones receiving corn oil. But, no substantial global difference between the two groups was seen.

Because EPA metabolism in cell membranes results in less pro-inflammatory eicosanoids, namely 3-series prostaglandins and 5-series leukotrienes, than the respective 2-series and 4-series derived from AA metabolism, these omega-3 fatty acids have been considered anti-inflammatory [18]. The anti-inflammatory properties attributed to omega-3 PUFAs that are potentially responsible for healing articular structures, cells, and molecules whose metabolism is affected by OA include reduction of proteoglycan-degrading enzymes, COX-2, and inflammatory cytokines IL-1 and TNF-α, as shown in vitro [45]. There is a need to prove these effects in clinical studies with fish oil products used as nutraceutical compounds to treat OA of companion animals [62, 63] and humans [5, 39]. In the dogs whose samples were analyzed here, the evaluated clinical outcome measures reflecting joint function and pain improved in the group receiving fish oil, whereas only pain improved in the corn oil group [43].

Changes in the diagnostic markers of oxidation (MDA, NTBI and 8-OH-dG) indicate a reduced concentration of oxidation in the fish oil group and suggest a possible effect of EPA and DHA on oxidative damage in tissue lipids. EPA and DHA might have acted as antioxidants or boosted the endogenous antioxidant mechanisms. The exact mechanism of action by which fish oil can regulate the level of plasma and tissue diagnostic marker enzymes in OA condition is unclear. Despite scant knowledge based on in vivo models, it is believed that dietary omega-3 PUFA stabilizes cell membranes by modulating the lipid environment [64], thereby making it less susceptible to damage caused by inflammatory agents. Concerning the oxidative stress molecules involved in OA, our findings of decreased MDA serum concentrations in all dogs may indicate that a similar degree of protection against lipid peroxidation was conferred by both fish and corn oil. Osteoarthritis is believed to be caused by mechanical stress on the joint leading to low grade inflammatory processes [7]. ROS are key components of many normal physiological processes that, at moderate levels, act as indispensable second messengers. However, higher than normal intracellular ROS concentrations can still overpower the homeostatic proteins and cause oxidative damage to the cell and contribute to the onset and progression of OA by inducing chondrocyte death and matrix degradation [65]. It has been shown that MDA, as a marker of lipid peroxidation, is increased in inflamed cartilage tissue and synovial fluid [23, 28, 66] as well as in blood of humans [29, 67] and dogs [32] suffering from OA. In vitro assays have demonstrated cartilage degradation mediated by lipid peroxidation [8]. On the other hand, the possibility of a heightened risk of lipid peroxidation after using fish oil has also been emphasized in a recent review [18], but was not confirmed here. Another study has demonstrated that fish oil decreased MDA levels in healthy dogs [68]. The slight elevation in values of 8-OH-dG that we found in both fish and corn oil groups was non-significant and fell below the reported plasma values for healthy dogs [69]. It indicates that during the study period the dogs did not exhibit evidence of DNA damage detected by measuring 8-OH-dG either before or after receiving fish or corn oil. In humans, elevations of 8-OH-dG have been reported in diverse conditions, including rheumatoid arthritis [70]. However, some authors consider 8-OH-dG measurement from the blood to be a questionable marker of oxidative stress, emphasizing the need for more research [71]. We suggest that the same is true for veterinary medicine, given the scarcity of reports on 8-OH-dG measurements in dogs. In any case, the results described herein contribute to the research, confirming that supplementation with fish oil, and even with corn oil, is significantly associated with decreased concentrations of lipid peroxidation products, namely MDA, in the plasma of OA dogs. Moreover, no evidence for lipid peroxidation emerged with the combined assays used in this study. Other authors have demonstrated that fish oil, which is rich in omega-3 PUFA, decreases the production of inflammatory series of prostaglandins and cytokines [72]. Our results indicate reduced oxidative stress markers in OA dogs supplemented with dietary oils, perhaps involving an equivalent mechanism, boosting the antioxidant defence in vivo. Thus, it can be assumed that dietary fish oil might enhance the antioxidant capability by inhibiting the formation of pro-inflammatory mediators or by upregulating the activities of other enzymes directly or indirectly associated with the antioxidant system.

The reduction of serum free iron that we found in the corn oil group (significant reduction) and in the fish oil group (non-significant) suggests at least a less favourable metabolic environment for oxidative damage. Presence and accumulation of iron has been reported to facilitate the formation of ROS, such as the hydroxyl radical, which is the most important radical in 8-OHdG production [54, 73]. The forms of free iron include non-transferrin bound iron (NTBI) [74]. An excess of intercellular free iron causes cell damage by catalysing the production of the hydroxyl radical through the Fenton reaction [75, 76]. Thus, excessive free iron could be a reliable parameter of iron toxicity [75, 77]. Evidence has suggested that oxygen free radicals, especially ones promoted by free iron, play an important role in the development of diseases [75, 77]. Free iron can damage tissues by catalysing the conversion of hydrogen peroxide to free radical ions that attack lipids, proteins and DNA [75, 78]. Fish and corn oil, as seen in the present study, may have played a protective role against increased free iron, possibly through iron binding and export, thus preventing iron-induced toxicity via the Fenton reaction [76].

The results obtained for blood measurements of GSH and Free-Car indicate that both oils were capable of maintaining the integrity of the antioxidant defence system. Moreover, fish oil supplementation induced a significant rise in blood concentrations of Free-Car. L-carnitine protects cells against oxidative damage since it acts as a free radical scavenger and has an important function in lipid metabolism by transporting fatty acids across the inner mitochondrial membrane [79]. Abundant evidence indicates the importance of GSH in maintaining the antioxidant homeostasis in cartilage and during articular pathological conditions [23, 27, 29, 67, 80–83]. Glutathione is a tripeptide (y-Glutamyl-cysteinyl-glycine) thiol present in virtually all animal cells – in normal conditions, both the reduced (GSH) and oxidized (GSSG) forms of glutathione remain in a balanced state [53]. GSH acts as a free radical scavenger and neutralizes superoxides, peroxide radicals and singlet oxygen by donating hydrogen atoms [84]. Antioxidant enzymes like glutathione S-transferase (GST) and glutathione peroxidase (GPX) utilize reduced forms of GSH to carry out their scavenging and detoxification activities, and at the end of the process, an oxidized form of GSSG is the final product [85]. Kumar and Das [86] reported a higher concentration of glutathione in rats with Freund’s adjuvant (CFA)-induced inflammation that were treated with fish oil. Arab et al. [87] noted that DHA enhanced the cellular GSH concentration by elevating the activities of gamma-glutamyl-cysteinyl ligase and glutathione reductase enzymes. PUFAs were reported to enhance the activity of glutathione reductase [88] and glucose-6-phosphate dehydrogenase enzymes [89], which in turn can restore GSH at a faster rate.

So far, a number of clinical studies have shown systemic evidence of a reduced antioxidant defence system, including reduced blood concentrations of GSH, in the course of inflammatory articular diseases in humans [90–93]. Although the blood values for GSH were within the reference values for dogs [94] before the trial in dogs with OA, we observed elevated values after 16 weeks of oil supplementation. Thus, we attribute these elevations to the supplementation with either fish oil or corn oil. The increase in GSH may be beneficial since it was concurrent with a reduction of MDA in both groups of dogs. In fact, it has been shown in dogs that GSH has an important role in adequate lipid peroxide detoxification during illness [94]. In the literature on canine OA, however, the only report on blood markers of oxidative stress associated with the disease was in a study using an experimental Pond-Nuki model [30]. As far as we know, no studies exist on oxidative and antioxidant parameters in naturally occurring canine OA, apart from the results reported here. Moreover, our study shows inedited results of the effect of supplementation with fish and corn oil on the oxidative/antioxidant balance in natural occurring canine OA. Nonetheless, further research should include analyses of serum isoprostane and prostaglandin as markers of oxidation and inflammation. In our study, limited biological samples were available.

The results of blood biochemistry, all within the normal range, support the conclusion that 16-week supplementation with fish or corn oil was not associated with toxicity or metabolic imbalance in dogs. Although hyperglycaemia was listed as a potential adverse effect of fish oil supplementation of dogs [18], we found no evidence of such an effect. In fact, the glucose concentrations were significantly higher within the corn oil group after 16 weeks of supplementation, although no significant difference was seen between the two groups. Moreover, all values remained within the reference range. Similarly, the cholesterol concentrations were significantly higher in the dogs after receiving corn oil, but not fish oil, but at the end of the 16-week trial they remained within normal reference values. These are in tune with a recent study where fish oil supplementation increased plasma triglycerides and ghrelin but did not appear to affect protein metabolism or postprandial glycaemia in adult lean dogs, whereas an increase in cholesterol concentration could be seen in the control diet group [95].

Concerning haematology tests, most values stayed within references for the dogs in our study. Administration of fish or corn oil during 16 weeks caused no change in blood haemoglobin concentration, although significant elevations of MCH and MCHC were seen in both groups from baseline to the end of the trial. The elevation of haemoglobin concentration within cells in both groups of dogs may be attributed to the fact that the mean values were at the lower end of the reference range at the beginning of the study. Thus, the oils seem to have enhanced haemoglobin synthesis towards normality during the 16-week study. There are no similar or comparable reports in dogs in the literature. In humans, there are reports of haemoglobin reduction in healthy individuals [96] and no change in patients with risk of anaemia after supplementation with fish oil [97, 98]. Likewise, we observed a significant reduction of the MCV values within the fish oil group, but comparable changes in dogs were not found elsewhere; also, no statistical difference was observed between groups. Elevations in packed cell volume after ingestion of fish oil for six weeks have been reported in healthy human beings [96], and no difference were found in patients with risk of anaemia [97]. The leucocyte counts of dogs in the present study revealed a significant reduction in the number of monocytes and basophils in the group receiving fish oil, from baseline to the end of the trial. These changes were also significant in the comparison with the dogs receiving corn oil over the same period. However, all counts, including those for other white cells, remained within reference values for dogs. Changes in eicosanoid expression towards the generation of leukotriene B5 induced by EPA ingestion have been speculated not to promote differences in circulating leucocyte counts in humans [99], although at least one study has shown increases in total leucocyte and monocyte counts after fish oil supplement [96]. It has also been shown that omega-3 fatty acids are able to interfere with leucocyte chemotaxis, adhesion molecule expression and leucocyte-endothelial adhesive interactions [60], the effect of which, attributed to the decrease of AA content of cells involved in immune responses, is associated with clinical improvement of inflammatory diseases including rheumatoid arthritis [100]. Even though a higher ratio of leukotriene B5/B4 has been reported in dogs supplemented with fish oil than in dogs given corn oil [17], no information on leucocyte counts in studies with fish oil supplementation of healthy dogs or dogs with OA was earlier available in the literature. A clinical study in dogs has, however, shown that the concentration of leukotriene B4 in osteoarthritic hip joint capsules was higher than in clinically normal ones [101]. Still, extensive and recent reviews on the therapeutic use of fish oil for animal diseases, including OA, do not mention its impact on haemograms [62].

The platelet counts in the present study showed that supplementation with corn oil resulted in lowered circulating numbers from the beginning to the end of the 16-week trial and that fish oil ingestion was associated with a non-significant elevation of platelets during the same period. Nevertheless, the values remained within the normal ranges for dogs in both groups, and the comparison between groups at the end of the trial showed negligible and non-significant differences. No bleeding events occurred during the clinical follow-up of the dogs. Although altered platelet function has been considered a potential risk of fish oil supplementation in dogs, a recent review concluded that there is no evidence of a harmful effect on platelet function or counts or clinical consequences due to fish oil or its fatty acids [18].

From our observations of both dog groups, we can hypothesize that the oils, particularly the fish oil given to the dogs as a supplement seemed to have promoted an abundance of beneficial polyunsaturated DHA and EPA, thus altering the availability of certain fatty acids in the body. At the cell membrane level, these fatty acids could have facilitated the repair of mitochondrial and other membranes, which could have been damaged by ROS due to inflammation in OA. As depicted by the significantly elevated concentrations of carnitine and reduced concentrations of MDA within the group of dogs that received fish oil, these fatty acids may also have promoted a better state of redox regulation. Clinically, the favourable shift in the membrane composition of lipids would be related to the reduction of pain and lameness indicators described in the dogs [43]. As OA often has a neuropathic aspect when chronic, the ROS contribution might be even more pronounced clinically at later stages: Neurons are especially sensitive to ROS since neurons have greater energy demands to function as compared to glial and other cells in the central nervous system [102, 103]. Also lipid peroxidation products may contribute to neuropathic pain in OA as they have been shown to contribute to neuropathic pain in chronic spinal cord injury animals [104]. However, the mechanism that ROS and lipid peroxidation play in chronic neuropathic pain is not well understood.

When the organism has more beneficial fatty acids it becomes less prone to peroxidation. For therapeutic use, a recent review has discussed and clarified the basis for what the authors called Lipid Replacement Therapy, in which membrane lipids can be replaced by, among others, oral supplements, promoting therapeutic effects on different diseases [105]. As well, Scicchitano et al. (2012) have reviewed and proposed an important role for nutraceuticals, including fish oils, in the control of lipid metabolism thus improving the overall burden of oxidation of lipids [106]. In the present study, fish oil supplementation seems to have promoted such effects, and corn oil to a lesser extent [43]. Considering that OA is a chronic disease whose clinical presentation takes longer than the actual onset of pathological symptoms, our results suggest that a longer follow-up would maybe have allowed clearer differences to emerge. In the future, the extent to which fish oil modifies the redox balance in healthy dogs and dogs with OA should be evaluated. Future fish oil supplementation study groups should also be stratified according to if they have a neuropathic aspect of pain. Further, one should consider another control substance than corn oil.

## Conclusion

In global terms, there was no clear difference between dogs fed fish oil or corn oil. The oxidative status markers indicated a decrease in oxidative stress (MDA and iron) and an increase in antioxidant capacity (Free-Car and GSH) both in dogs receiving fish oil and corn oil. The elevation of the antioxidant capacity and the decrease of inflammatory monocytes and basophils were, however, more significant in the fish oil group and are unprecedented results reported in dogs with OA. Despite the fact that values remained within the normal range, cholesterol and glucose increased significantly only in the corn oil group.

## Abbreviations

8-OH-dG: 8-hydroxy-2-deoxyguanosine
AA: Arachidonic acid
ALA: α-linolenic acid
ALP: Alkaline phosphatase
ALT: Alanine aminotransferase
ANOVA: Analysis of variance
BW: Body weight
CAT: Catalase
CFA: Complete Freund’s adjuvant
CONSORT: Consolidated standards of reporting trials
COX: Cyclooxygenase
DHA: Docosahexaenoic acid
DHGLA: Dihomo-gamma-linolenic acid
DMARD: Disease-modifying anti-rheumatic drug
DPA: Docosapentaenoic acid
EPA: Eicosapentaenoic acid
ETA: Eicosatetraenoic acid
FA: Fatty acid
FFA: Free fatty acid
Free-Car: Free carnitine
GLA: Gamma-linolenic acid
GPX: Glutathione peroxidase
GSH: Reduced glutathione
GSSG: Oxidized glutathione
GST: Glutathione S-transferase
HCPI: Helsinki chronic pain index
HPLC: High-performance liquid chromatography
IL-1: Interleukin-1
LA: Linoleic acid
MCH: Mean Corpuscular Haemoglobin
MCHC: Mean Corpuscular Haemoglobin Concentration
MCV: Mean Corpuscular Volume
MDA: Malondialdehyde
MMP: Matrix metalloproteinase
NO: Nitric oxide
NSAID: Non-steroidal anti-inflammatory drug
NTBI: Non-transferrin bound iron
OA: Osteoarthritis
OLA: Oleic acid
PA: Palmitic acid
PUFA: polyunsaturated fatty acid
PVF: Peak vertical force
RA: Rheumatoid arthritis
ROS: Reactive oxygen species
RV: Reference values
SD: Standard deviation
SDA: Stearidonic acid
SOD: Superoxide dismutase
TBARS: Thiobarbituric acid reactive substance
TIMP: Tissue inhibitor metalloproteinase
TNF-α: Transforming growth factor alpha
TP: Total protein
W0: Baseline
Wx: x weeks after baseline
W-x: x weeks before baseline
ω: Omega
